# The role of psychological factors on improving work engagement among nurses

**DOI:** 10.3389/fpsyg.2024.1419855

**Published:** 2024-08-27

**Authors:** Hasan Abualruz, Ahmad Rayan, Suhair Al-Ghabeesh, Mirna Fawaz, Rayan Jaafeer, Batool Qutami, Hanan Alyami

**Affiliations:** ^1^Faculty of Nursing, Al-Zaytoonah University of Jordan, Amman, Jordan; ^2^Faculty of Nursing, Zarqa University, Zarqa, Jordan; ^3^College of Health Sciences, American University of the Middle East, Kuwait City, Kuwait; ^4^Nursing Department, Faculty of Health Sciences, Beirut Arab University, Beirut, Lebanon; ^5^Department of Basic Medical Sciences, Faculty of Medicine, Al-Balqa Applied University, Al-Salt, Jordan; ^6^Department of Medical and Surgical Nursing, College of Nursing, Princess Norah Bint Abdurrahman University, Riyadh, Saudi Arabia

**Keywords:** emotional intelligence, resilience, psychological empowerment, critical care nurses, work engagement

## Abstract

**Background:**

Work engagement is affected by many psychological variables including emotional intelligence, psychological empowerment, and resilience that are not well-studied among nurses.

**Purpose:**

This study aims to examine the impact of emotional intelligence on the work engagement of critical care nurses, and the mediating role of resilience and psychological empowerment.

**Methods:**

A descriptive cross-sectional design was adopted in this study among 150 critical care nurses at one university hospital in Saudi Arabia. Independent *t*-test and correlational analysis were used to assess relationships between study variables. A multi-step regression model was used to assess the mediatory effect.

**Results:**

The results showed that a statistically significant positive association exists between each of the study variables (*p* < 0.01). The regression model showed that higher resilience (*p* < 0.001) and psychological empowerment (*p* < 0.001) predicted higher work engagement. The model predicted 33.3% of the changes in work engagement scores among critical care nurses.

**Conclusion:**

To enhance work performance and quality of care rendered at critical care units, higher emphasis should be placed on emotional intelligence and other significant psychological variables.

## 1 Introduction

The healthcare quality provided by nurses depends on their mental health, achievements, and levels of involvement. The demanding circumstances in which they perform their nursing duties lead to work-related stress, in turn leading to detrimental effects on psychological and physical health, and eventually negatively affecting their engagement at work (Giménez-Espert et al., 2020).

Nurse shortages, work overburden, low wages, mismanagement, toxic working environments, and workplace abuse are common problems in the healthcare sector. These issues give rise to feelings of worry, distress, and anxiety among nurses, which can lead to feelings of tension and uneasy mental state that may negatively affect employees’ ability to be involved (Subih et al., 2023).

Abualruz et al. (2023) expressed that nurses endure high degrees of work-related stress because of their own personal and social circumstances along with risks posed by constant contact with patients. Nurses are constantly subjected to patients’ suffering such as death, and sadness, and their problems can cause them to feel mentally worn out. As a result, frontline workers must endure the emotional management of dealing with their negative feelings as well as those of others.

Work engagement has been identified as an essential part of forming favorable organizational outcomes. Encouraging engagement empowers nurses, leading to better patient care. In the same perspective, Carthon et al. (2019) stated nurses’ engagement is linked to decreased mortality rates as well as increased profits and success in the business sense. Although work engagement is necessary for organizational success, McCormack (2021) found that engagement levels among employees are gradually decreasing. Scholars have explained the reason behind the decrease in nurses’ engagement might be due to unsuccessful mechanisms that are implemented to make employees more engaged.

To improve efficiency and engagement among healthcare employees, Chikobvu and Harunavamwe (2022a,b) proposed that companies should focus on strengths and individual assets. Prins et al. (2018) found that emotional intelligence and other internal psychological personal resources could empower employees to have a better attitude at work by reducing the effects of external stimuli on work engagement. Additionally, Danquah (2022) noted that internal psychological motivators aid individuals in evaluating themselves, enabling them to better control and well adaptation to their circumstances and improve work engagement. As a result, they confront goals, which leads to work achievements. Emotional intelligence, resilience, and psychological empowerment are the most common internal resources. Few studies in the literature targeted the impact of these factors on nurses’ engagement to work globally. In the Arabic region, this study is the first of its kind. Therefore, it is important to fill this gap in the literature and lessen the problem of nurse turnover.

The study intended to determine whether emotional intelligence and resilience influence work engagement and psychological empowerment among nurses at Al Moussa Hospital in Saudi Arabia, by addressing the following hypothesis:

Hypothesis 1: Emotional intelligence has a positive influence on work engagement.

Hypothesis 2: Resilience and psychological empowerment mediate the relationship between emotional intelligence and work engagement.

## 2 Literature review

### 2.1 Work engagement

Work engagement is complex concept that has been introduced in the literature as motivational state of satisfaction, dedication, and absorption (Ghazawy et al., 2021). It was first developed to transform the focus from employees’ unhappiness to well-being in burnout literature. Zammitti et al. (2022) determined that work engagement among nurses is an optimistic and satisfying work-related state of mind which is signalized by the ability to fully concentrate on one’s job as well as high energy and enthusiasm levels during the job. It has been reported in the literature that work engagement is essential in demanding work environments such as hospitals (Allande-Cussó et al., 2021), as it positively reflected on employees satisfaction, commitment, happiness, and thus the quality of care and patient’s safety (Ghazawy et al., 2021).

Work involvement has been shown to have benefits for both the individual and the organization since it increases employee motivation and commitment (El-Gazar et al., 2022). Weideman and Hofmeyr (2020) reported that work engagement positively affects occupational health and increases revenue for organizations as well as leading to lower absence, a safe work environment, and empowered employees. Despite the benefits of employee involvement, Naidoo et al. (2019) brought up that 64% of employees worldwide are not involved. Therefore, it is necessary to develop ways that enhance work involvement. Demerouti et al. (2021) explained that work engagement could be boosted by widening personal resources.

### 2.2 Emotional intelligence

In the nursing context, emotional intelligence is the ability to comprehend the emotions of patients through proper communication as well as using appropriate words and actions. Lu et al. (2022) introduced the concept of emotional intelligence as a person’s ability to control feelings and direct them to control behaviors and thoughts. Nurses are usually highly emotional workers, due to their nature of work. Therefore, the study of emotional intelligence is becoming more prevalent in nursing. Having emotional intelligence aids nurses when it comes to dealing with stress, making better decisions, handling emotions effectively, and positively affecting their care of patients as well as their health (Christianson et al., 2021). Ishii et al. (2015) discussed that emotional intelligence helps nurses to reflect on their experience in predicting unexpected outcomes in practice.

A study that assessed the role of EI in engagement in nurses showed that nurses with higher levels of EI also had higher levels of engagement (Pérez-Fuentes et al., 2018). Effective EI training may be a crucial strategy to prevent nurses from quitting their jobs, decreasing stress, and decreasing burnout (Codier and Codier, 2017).

### 2.3 Resilience

Resilience is both a mental and behavioral process that involves the use of personal resources and shielding oneself from the negative aspects of difficult situations. Therefore, to build resilience, one must have the ability to manage their immediate environment and develop protective measures. To ensure positive adaption, one must view resilience as a beneficial trait instead of just a means to deal with stress (Abualruz and Hayajneh, 2023).

Resilience varies from one person to another; therefore, it is an individual’s flexibility capability. These are considered internal resources that decrease the effect of the job. Work-related achievements are positively influenced by resilience-such as work engagement (Meintjes and Hofmeyr, 2018).

### 2.4 Psychological empowerment

Psychological empowerment is a response to the work environment which may be psychological, cultural, or social. It can be used to control, express, and satisfy their needs as well as decide about their feelings (Fan et al., 2016). It is described by four pillars: (1) meaning, (2) competence, (3) self-determination, and (4) impact. Psychological empowerment reduces work-related stressors and burnout (Gong et al., 2020).

The definition of empowerment varies across different cultures. It is agreed that in empowerment, employees take part in decision-making, high-quality care providers, and agency goal achievers. Empowerment involves freedom of choice, the ability to act, and the capability to decision making (Orgambídez-Ramos et al., 2017). Therefore, employees need to have the necessary information, support, feedback, and guidance from superiors, and co-workers to achieve their work correctly (Saleh et al., 2022).

### 2.5 Emotional intelligence and work engagement

Various research has shed light on the effects of EI on work engagement (Extremera et al., 2018). As mentioned earlier, the Job Demands-Resources model declared that internal resources are very important for work engagement. Workers who are engaged report more positive emotions such as enthusiasm, happiness, and satisfaction. Furthermore, studies have concluded that there is an effect of work engagement on personality and organizational goals, such as job performance and career satisfaction (Giménez-Espert et al., 2020).

### 2.6 Emotional intelligence and psychological empowerment

Emotional intelligence is an important motivational resource and is thought to increase the engagement of employees with their work (Meng and Sun, 2019). Furthermore, personality, such as self-esteem, was established as a crucial role in psychological empowerment (Farčić et al., 2020). Therefore, a major antecedent of psychological empowerment was presumed to be emotional intelligence.

Those with higher psychological empowerment demonstrated higher intrinsic need fulfillment in their jobs which leads to higher job satisfaction. Research indicated that psychological well-being is a mediator of work context and outcomes (Akkoç et al., 2022). A significant amount of research has shown that for psychological empowerment, the trait of emotional intelligence is necessary (Sun et al., 2017).

### 2.7 Emotional intelligence and resilience

Magnano et al. (2016) explained the positive effects of emotional intelligence on work involvement are strengthened by resilience. Controlling emotions in oneself and others is necessary in lowering negative evaluations, thus increasing the ability to manage conflicts in the form of flexibility. Therefore, it can be argued that resilience motivates endurance of obstacles and promotes positive states (and challenge orientation; Meintjes and Hofmeyr, 2018). In turn, behaviors of resilience are thought to have a notable influence on individuals’ levels of engagement. In this study, the researcher has assessed the relationship between emotional intelligence oand work engagement among nurses and tested the mediation effect of resilience and psychological empowerment on the relationship.

**Table:** 

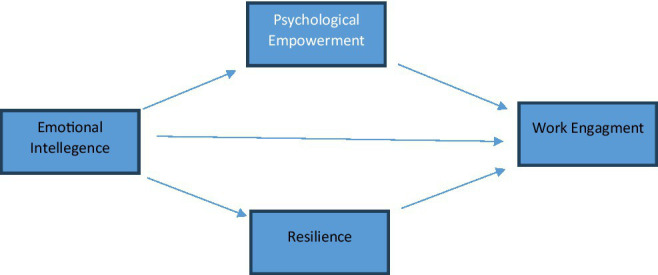

## 3 Methodology

### 3.1 Design

This research was designed to be a cross-sectional study to ascertain the mediating effect of resilience and psychological empowerment on emotional intelligence and work engagement among critical care nurses.

### 3.2 Setting

This study was conducted among nurses working at Al Moussa Hospital in Saudi Arabia. Al-Moosa Specialist Hospital is a private medical facility in the city of Al-Ahasa, Eastern Province of Saudi Arabia. With a total bed capacity of 240, the hospital is considered among the finest in the area. It offers the widest range of medical services as well as highly qualified medical, nursing, and paramedical staff and highly advanced medical equipment.

### 3.3 Population and sampling

The sample of this study includes nurses working in the critical care areas of Al Moussa Hospital. The sample was recruited from ICU wards at the hospital. The study employed a convenience sample where critical care nurses who have been working at Al Moussa Hospital, met the eligibility criteria (Nurses working in intensive units for more than 6 months), and agreed to voluntarily participate in the study were included in the sample. A convenience sample is a technique with no statistical probability, which involves the researcher integrating all probable respondents who are met in the selection process.

### 3.4 Tools

#### 3.4.1 Demographic characteristics

A demographic questionnaire including the participant’s age, gender, years of experience, and other characteristics was used.

#### 3.4.2 Emotional intelligence

Wang and Law’s Emotional Intelligence Scale was adopted to assess the level of emotional intelligence (Wong and Law, 2002). The scale has a total of 16 items and 4 dimensions: “self-emotional perception,” “emotion regulation,” “emotional use,” and “recognize other’s emotions.” All items are rated on a Likert scale, with “1” standing for “strongly disagree” and “7” standing for “strongly agree.” The higher the total score on the scale, the higher the emotional intelligence level of nurses. The scale was valid and reliable with a Cronbach’s α coefficient of 0.926. The Cronbach’s α coefficient of the scale was calculated among the participants in this study and it was 0.91.

#### 3.4.3 Utrecht work engagement scale (UWES)

The 17-item UWES by Schaufeli and Bakker (2004) was employed to assess work engagement. Responses were scored on a 6-point Likert scale ranging from ‘0’ never to ‘6’ always. Schaufeli and Bakker (2004) revealed an acceptable reliability coefficient for the scale (α = 0.86). The higher the total score on the scale, the higher the work engagement among nurses. The Cronbach’s α coefficient of the scale was calculated among the participants in this study and it was 0.84.

#### 3.4.4 Wagnild and young resilience scale

The resilience scale RS-25 (Gail Wagnild, 2009) was employed to measure nurses’ resilience. The scale includes 25 items making up five components of the scale, namely perseverance, equanimity, self-reliance, existential aloneness, and meaningfulness. All items were scored on a 7-point Likert scale that ranged from 1 = disagree to 7 = agree. Koen et al. (2011), in a South African sample, revealed adequate reliability scores for the scale with a coefficient alpha of 0.80 and test–retest coefficients of 0.70–0.98. The higher the total score on the scale, the higher resilience among nurses. The Cronbach’s α coefficient of the scale was calculated among the participants in this study and it was 0.77.

#### 3.4.5 Psychological empowerment

Psychological empowerment was measured with a 12-item scale (Spreitzer, 1995) to evaluate individual views of psychological empowerment. This scale includes four subscales: meaning, competence, self-determination, and impact (e.g., “I have significant influence over what happens in my department.”). Participants answered on a five-point Likert scale ranging from 1 (strongly disagree) to 5 (strongly agree). This tool has stated a good level of reliability and validity, the internal consistency of the total psychological empowerment was 0.90 (Dust et al., 2018). The Cronbach’s α coefficient of the scale was calculated among the participants in this study and it was 0.88.

### 3.5 Data collection

1. An IRB approval was obtained from the research department at Al Moussa Hospital where the study was conducted.
2. Informed consent including the details of the research study such as background information, the aim of the study, benefits of participation, confidentiality policy as well as the contact information of the researcher was also obtained from the participants.
3. The researcher provides information about the aim, content, and duration of the project that will be conducted and what participants are required to do.
4. Nurses who freely accepted to partake in the study filled out the questionnaires. The researcher approached the nurse in charge of the specific floors and asked for consent to distribute the tools during their meetings among the nursing staff before approaching nurses.

### 3.6 Ethical considerations

The following ethical requirements were taken into consideration during the study:

- Approval to carry out the study from the responsible authorities was secured.
- An informed consent was obtained from the nurses to collect the required data.
- The anonymity of the study subjects was maintained; no names were recorded during data collection and reporting.
- Data obtained was confidential and properly secured

## 4 Results

### 4.1 Participant characteristics

The sample of this study was made up of one hundred and fifty (*N* = 150) nurses, where 26 (17.3%) of them were males and 124 (82.7%) of them were females. The descriptive analysis also revealed that the mean age of the participants was 30.35 (±5.5 years), and the mean years of experience was 7.2 (±4.55 years) (Table 1).

**Table 1:** Participant characteristics.

Variable	Category	*N*	%
Gender	Female	124	82.7
	Male	26	17.3
Age	*M*	SD	
	30.35	5.5	
Years of experience	7.26	4.55	

### 4.2 Descriptive statistics of study variables

**Figure 1:**
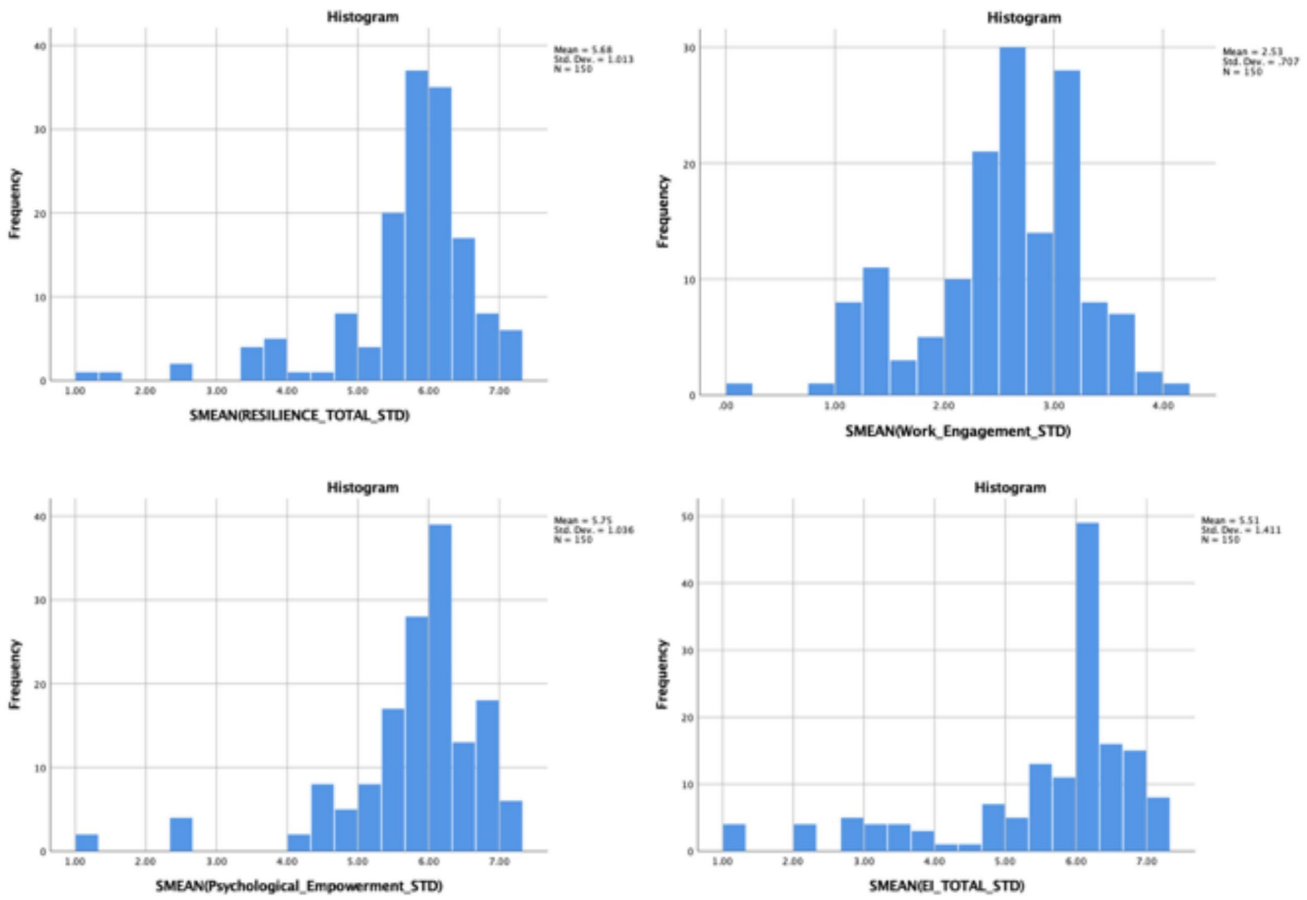
Distribution of standard scores of study variables inferential statistics.

Descriptive statistics were carried out to determine the mean total scores recorded by the study participants on the level of each study variable. The results showed that the participants reported a relatively high resilience mean standard score of 5.68 ± 1.01 (95% CI: 5.52, 5.85) with a small standard error (SE = 0.08). This was also the case with the emotional intelligence mean standard score of 5.51 ± 1.41 (95% CI: 5.28, 5.74), and the psychological empowerment mean standard score of 5.75 ± 1.04 (95% CI: 5.58, 5.91). As for work engagement, the participants reported an above-average mean standard score of 2.53 ± 0.71 (95% CI: 2.42, 2.64) (Table 2). The data is minimally skewed to the left; however, the assumption of normality is preserved due to the sample size (Figure 1).

**Table 2:** Total scores of study variables.

	Min	Max	Mean	SD	95% CI	SE
Resilience total score	1	7	5.68	1.01	5.52, 5.85	0.08
Emotional intelligence total score	1	7	5.51	1.41	5.28, 5.74	0.11
Psychological empowerment total score	1	7	5.75	1.04	5.58, 5.91	0.085
Work engagement total score	0	4	2.53	0.71	4.00	0.06

An independent *t*-test was carried out to determine if there is an association between study variables and gender. It was significant for work engagement and emotional intelligence raw total scores (*p* = 0.02, *p* = 0.01), respectively. The results of the t-test showed that there was an association between work engagement and gender, where females scored higher than males (*p* = 0.05) (Table 3).

**Table 3:** Association between gender and study variables.

	Gender	Mean	SD	*t*	95% CI		*p*-value
Resilience total score	Female	141.07	26.84	−1.08	−16.68	4.89	0.27
	Male	146.97	15.69	−1.51	−13.71	1.92	
Work engagement total score	Female	44.08	11.18	2.45	1.21	11.29	0.02
	Male	37.83	14.55	2.07	0.08	12.41	
Emotional intelligence total score	Female	89.99	20.33	2.16	0.87	19.89	0.01
	Male	79.61	30.20	1.67	−2.28	23.04	
Psychological empowerment total score	Female	68.45	13.07	−1.07	−8.15	2.44	0.13
	Male	71.31	8.57	−1.39	−6.97	1.26	

A bivariate Pearson’s correlation was carried out to determine if there is an association between study variables and age and years of experience of participants. The results showed that there is a statistically significant weak negative association between resilience and age (Table 4).

**Table 4:** Association between study variables, age, and experience.

		Age	Years of Experience
Resilience Total Score	R-value	−0.21	−0.02
	P-value	**0.01**	0.82
Work Engagement Total Score	R-value	0.00	−0.04
	P-value	1.00	0.60
Psychological empowerment total score	R-value	−0.03	0.10
	P-value	0.75	0.23
Emotional Intelligence Total Score	R-value	0.00	0.03
	P-value	1.00	0.73

**Figure 2:**
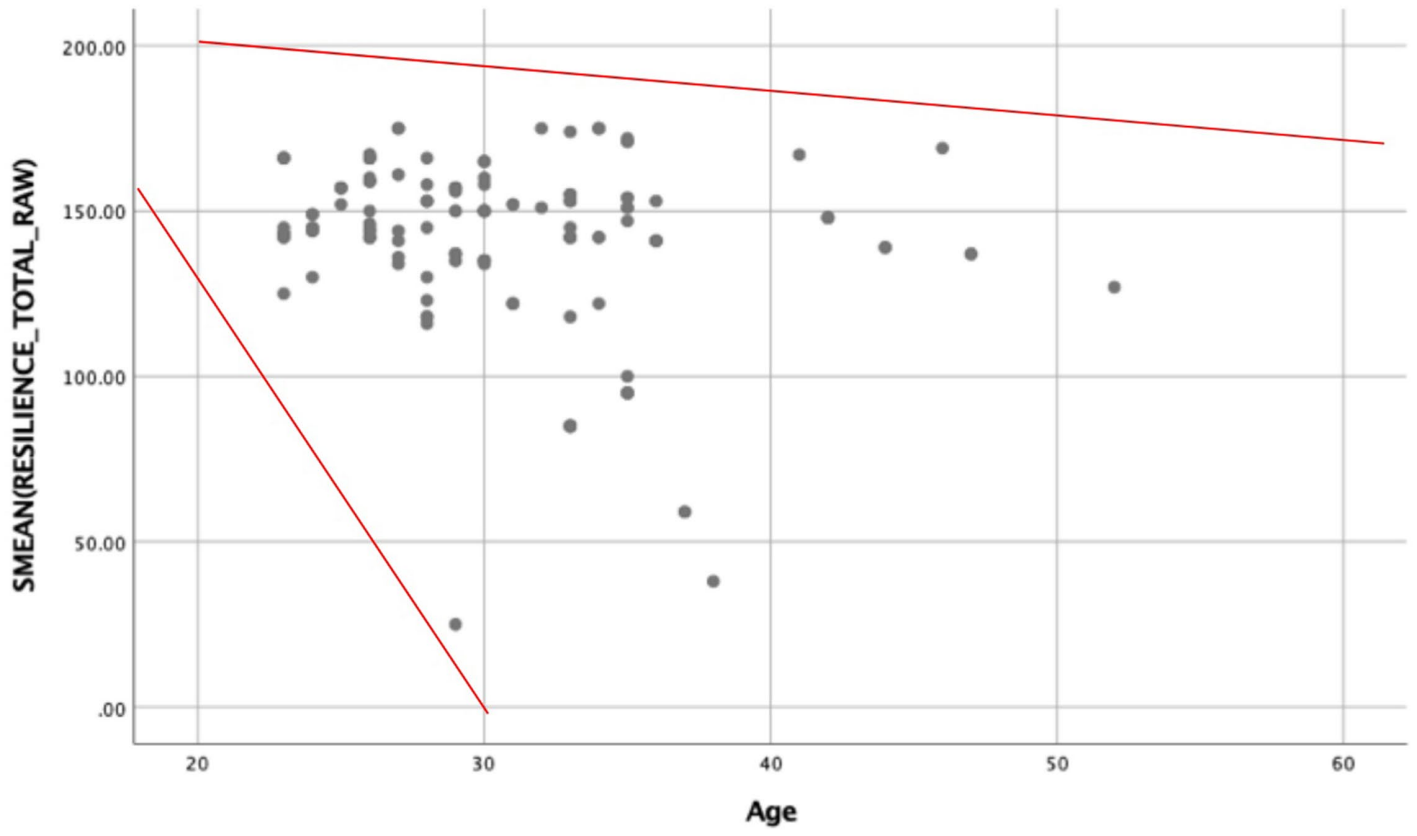
Association between age and resilience.

This weak association can be indicated by the following scatterplot (Figure 2).

Another Pearson’s bi-variate correlation was carried out to explore the possible associations between the study variables. The results showed that emotional intelligence, resilience, psychological empowerment, and work engagement among critical care nurses were statistically significantly and positively correlated with each other, where associations ranged from moderate to strong correlations (Table 5).

**Table 5:** Correlations between study variables.

		Resilience total score	Work engagement total score	Psychological empowerment total score	Emotional intelligence total score
Resilience Total Score	*R*-value	1.00	0.31^**^	0.85^**^	0.54^**^
	*P*-value		**<0.001**	**<0.001**	**<0.001**
Work engagement total score	*R*-value	0.31^**^	1.00	0.23^**^	0.55^**^
	*P*-value	**<0.001**		**0.01**	**<0.001**
Psychological empowerment total score	*R*-value	0.85^**^	0.23^**^	1.00	0.46^**^
	*P*-value	**<0.001**	**0.01**		**<0.001**
Emotional intelligence total score	*R*-value	0.54^**^	0.55^**^	0.46^**^	1.00
	*P*-value	**<0.001**	**<0.001**	**<0.001**	

Bold means significant *p* value. ** Significant at alpha 0.01.

Finally, to examine the mediatory role of resilience and psychological empowerment a multi-step regression model was conducted, where the dependent variable was set to be work engagement. First, a model including only the participants’ characteristics was conducted, where gender was found to be a statistically significant negative predictor of work engagement. The model showed that moving to the male gender reduces work engagement by *β* = 6.28 points (*p* = 0.02) while controlling for age and years of experience. However, this model only explains 4.4% of the variations in work engagement scores. The second model included emotional intelligence as an independent variable. The results showed that an increase in one point in emotional intelligence increases work engagement among critical care nurses by 0.29 points while adjusting for the other variables in the model (*p* < 0.001). The predicting value of gender was diminished in this step. This model, however, explains 32.6% of the variations in nurses’ work engagement. Finally, resilience and psychological empowerment were added to the regression model. The results showed that a one-point increase in resilience increases work engagement by 5.2 points (*p* < 0.001), and a one-point increase in psychological empowerment increases work engagement by 4.7 points (*p* < 0.001). This model explains 33.3% of the changes in work engagement scores among critical care nurses (Table 6).

**Table 6:** Regression models.

Variables	Model 1			Model 2			Model 3		
	*B*	*P*-value	SE	*B*	*P*-value	SE	*B*	*P*-value	SE
Gender	0.13	0.60	0.33	0.30	0.29	0.14	0.46	0.14	0.31
Age	−6.28	**0.02**	2.61	−3.08	0.17	−0.10	−3.27	0.15	2.28
Years of experience	−0.30	0.44	0.39	−0.49	0.15	−0.18	−0.61	0.09	0.35
Emotional intelligence	-	-	-	0.29	**<0.001**	0.54	0.26	**<0.001**	0.05
Resilience	-	-	-				5.20	**<0.001**	0.07
Psychological empowerment	-	-	-				4.70	**<0.001**	0.13
*F*	2.25	0.09		17.56	0.07		11.92	0.06	
*R*^2^	0.04			0.326			0.33		
Adjusted *R*^2^	0.03			0.308			0.31		
*R*^2^ change	--			0.286			0.004		

Bold means significant *p* value.

## 5 Discussion

According to the findings of our study, work engagement among critical care nurses was strongly and favorably correlated with emotional intelligence. Our findings are in line with other studies in the field (Gong et al., 2020), which found a favorable relationship between emotional intelligence and work engagement among registered nurses. Higher levels of work engagement are a reflection of nurses’ active participation, loyalty, and enthusiasm for their careers and are associated with higher emotional intelligence levels. The correlation between emotional intelligence and work engagement has merit for several reasons. The ability to understand and manage one’s own emotions as well as correctly perceive and respond to other people’s emotions is the first component of emotional intelligence. This improved emotional awareness and control may help nurses deal with challenging situations, manage stress, and maintain a good attitude, thus increasing their level of occupational engagement.

Awad and Ashour (2020) the investigators discovered that emotional intelligence was associated with work engagement among nurses who had been on the job for a longer amount of time, implying that emotional intelligence may become increasingly relevant for nurses as they acquire experience. This is inconsistent with our study results, where age was found to be a predictor of lower work engagement, and work experience was not found to be associated with better emotional intelligence, yet female gender was associated with higher work engagement. This might be attributed to the different cultural background among nurses who works in Sudi Arabi, whereas the majority of the nursing workforce are non-Saudi nurses.

Li et al. (2021) reported that emotional intelligence and work engagement were both connected to resilience and psychological empowerment in a group of 322 registered nurses. The findings of the study imply that emotional intelligence may be more crucial in assisting nurses in developing resilience because it was found to be more strongly connected with resilience than work engagement. This was confirmed by the results of our study, which demonstrated a substantial positive correlation between resilience, emotional intelligence, psychological empowerment, and work engagement.

This study’s results showed that resilience and psychological empowerment mediated the association between emotional intelligence and work engagement through multiple regression models. This is consistent with Chikobvu and Harunavamwe (2022a,b)who conducted a study on the association between emotional intelligence, resilience, and professional engagement in a sample of registered nurses. The findings demonstrated a statistically significant positive relationship between emotional intelligence and job engagement. In addition, resilience was shown to partially moderate this link. In a similar vein in the research conducted by AlZgool et al. (2020), the mediating role of resilience and psychological empowerment on the link between emotional intelligence and job engagement among nurses was reported as well.

### 5.1 Limitations

It is critical to recognize some of the current study’s shortcomings. First, because the study was cross-sectional, we were unable to show causal relationships between emotional intelligence and work engagement. Furthermore, the research relied on self-report measurements, which are susceptible to frequently occurring reporting and social desirability bias. Finally, the study was carried out in a specific healthcare environment, which may restrict the findings’ generalizability.

## 6 Conclusion and implication for nursing management

The findings of this study support the existence of a favorable relationship between emotional intelligence and work engagement among critical care nurses. Nurses with higher levels of emotional intelligence indicated higher levels of work engagement. Nurses who had greater emotional intelligence had more resilience and psychological empowerment, which affected their work engagement levels favorably. These results have consequences for healthcare organizations and nursing management. Recognizing the importance of emotional intelligence, resilience, and psychological empowerment in boosting engagement, healthcare organizations might prioritize initiatives to improve these traits among nurses.

To assist nurses in acquiring the needed skills and assets to successfully handle their emotions, overcome difficulties, and attain higher levels of work engagement, methods such as emotional intelligence training initiatives, resilience-building projects, and creating empowering workplaces can be put into effect. By concentrating on these factors, healthcare institutions may encourage nurses’ work engagement, leading to increased job satisfaction, well-being, and superior care provision.

## Data Availability

The raw data supporting the conclusions of this article will be made available by the authors, without undue reservation.
